# Oxidative Stress Disrupts Gill Function in *Eriocheir sinensis*: Consequences for Ion Transport, Apoptosis, and Autophagy

**DOI:** 10.3390/antiox14080897

**Published:** 2025-07-22

**Authors:** Wenrong Feng, Qinghong He, Qiqin Yang, Yuanfeng Xu, Gang Jiang, Jianlin Li, Jun Zhou, Rui Jia, Yongkai Tang

**Affiliations:** 1 Key Laboratory of Freshwater Fisheries and Germplasm Resources Utilization, Ministry of Agriculture and Rural Affairs, Freshwater Fisheries Research Center, Chinese Academy of Fishery Sciences, Wuxi 214081, China; fengwenrong@ffrc.cn (W.F.); hqh13990750864@163.com (Q.H.); xuyuanfeng@ffrc.cn (Y.X.); jianggang@ffrc.cn (G.J.); lijl@ffrc.cn (J.L.); jiar@ffrc.cn (R.J.); 2 Wuxi Fisheries College, Nanjing Agricultural University, Wuxi 214081, China; 3 Fishery Technology Extension Station of Yunnan, Kunming 650034, China; ynsctgk@163.com; 4 Freshwater Fisheries Research Institute of Jiangsu Province, Nanjing 210017, China; finedrizzle@163.com

**Keywords:** *Eriocheir sinensis*, oxidation, osmoregulation and ion regulation, MAPK, AMPK

## Abstract

Oxidative stress is a key mediator of physiological dysfunction in aquatic organisms under environmental challenges, yet its comprehensive impacts on gill physiology require further clarification. This study investigated the molecular and cellular responses of *Eriocheir sinensis* gills to hydrogen peroxide (H_2_O_2_)-induced oxidative stress, integrating antioxidant defense, ion transport regulation, and stress-induced cell apoptosis and autophagy. Morphological alterations in the gill filaments were observed, characterized by septum degeneration, accumulation of haemolymph cells, and pronounced swelling. For antioxidant enzymes like catalase (CAT) and glutathione peroxidase (GPx), activities were enhanced, while superoxide dismutase (SOD) activity was reduced following 48 h of exposure. Overall, the total antioxidant capacity (T-AOC) showed a significant increase. The elevated concentrations of malondialdehyde (MDA) and H_2_O_2_ indicated oxidative stress. Ion transport genes displayed distinct transcription patterns: Na^+^-K^+^-2Cl^−^ co-transporter-1 (*NKCC1*), Na^+^/H^+^ exchanger 3 (*NHE3*), aquaporin 7 (*AQP7*), and chloride channel protein 2 (*CLC2*) were significantly upregulated; the α-subunit of Na^+^/K^+^-ATPase *(NKA*α) and carbonic anhydrase (*CA*) displayed an initial increase followed by decline; whereas vacuolar-type ATPase (*VATP*) consistently decreased, suggesting compensatory mechanisms to maintain osmotic balance. Concurrently, H_2_O_2_ triggered apoptosis (*Bcl2*, Caspase-3/8) and autophagy (beclin-1, *ATG7*), likely mediated by MAPK and AMPK signaling pathways. These findings reveal a coordinated yet adaptive response of crab gills to oxidative stress, providing new insights into the mechanistic basis of environmental stress tolerance in crustaceans.

## 1. Introduction

In the aquatic environment, stressors such as fluctuations in salinity, alkalinity, temperature, dissolved oxygen levels, and ammonia nitrogen trigger defensive responses in organisms [1,2,3]. These responses are commonly associated with oxidative stress, which arises when reactive oxygen species (ROS) production overwhelms the detoxification capacity of antioxidant defenses [4]. In crustaceans, the gills are multifunctional organs that directly interface with the external environment, playing critical roles not only in gas exchange but also in osmoregulation and immune defense. However, the systemic effects of oxidative stress on gill physiology, including the interplay among antioxidant defense, ion homeostasis, and programmed cell death (such as apoptosis and autophagy), require further discussion.

Excessive levels of ROS can be harmful, leading to lipid peroxidation, protein misfolding, DNA breaks, and damage to cellular structures, which ultimately trigger cell death processes such as apoptosis, autophagy, or necrosis [5,6]. To counteract these harmful effects, organisms activate intricate antioxidant defense mechanisms. Key among these are antioxidant enzymes, including superoxide dismutase (SOD), catalase (CAT), and glutathione peroxidase (GPx), which collaboratively scavenge ROS and restore redox homeostasis [7].

Furthermore, the ion transport systems in gill tissues, which function in maintaining osmoregulation, calcium homeostasis, ammonia excretion, and extracellular pH regulation [8,9], may undergo significant effects under oxidative stress. Many studies on crustacean species have demonstrated gill damage resulting from exposure to salinity and toxic substances. Specifically, the impact of salinity on Na^+^/K^+^ ATPase (NKA) and antioxidant enzyme activities in the gills has been well documented [10,11]. Metals have been demonstrated to alter gill structures, hemolymph osmolality, and NKA in a variety of freshwater and marine crustaceans [12,13]. Pesticides can disrupt both respiration and osmoregulation in gill tissues, accompanied by severe oxidative stress, significantly affecting the health and survival of crustaceans [14,15]. Our study focuses on elucidating gene transcription profiles that mediate compensatory regulation of ion transport systems under oxidative stress conditions.

Concurrently, ROS-induced autophagy and apoptosis may serve as either cellular repair mechanisms or pathways to damage [16,17]. Excessive ROS induce apoptosis by activating the caspase cascade system, which is responsible for the induction, transmission, and amplification of apoptosis signals [18]. For example, intracellular stress like oxidative stress activates proapoptotic proteins in the B cell lymphoma 2 (Bcl2) family, including BAX and BAK [19]. This activation triggers mitochondrial outer membrane permeabilization (MOMP), leading to cytochrome c release, ultimately activating the caspase cascade. Additionally, ROS activate the tumor suppressor p53 [20], which subsequently promotes the expression and activity of proapoptotic proteins, such as BAX, thereby advancing the apoptotic process. ROS also serve as important regulators of autophagy. A key mechanism involves ROS-mediated activation of AMPK, which phosphorylates and inhibits mTOR complex 1 (mTORC1) to initiate autophagosome formation [21]. High ROS levels upregulate beclin-1, a critical autophagy regulator, leading to enhanced autophagosome formation [22]. Despite their known individual roles, how oxidative stress coordinates apoptosis and autophagy in crustacean gill tissues—and whether they function synergistically or antagonistically—remains to be elucidated.

Hydrogen peroxide (H_2_O_2_) is endogenously produced in cells through multiple pathways, including the mitochondrial electron transport chain at complex I/III, NADPH oxidase (NOX)-mediated superoxide conversion, and peroxisomal β-oxidation reactions. However, environmental stressors promote excessive H_2_O_2_ generation via oxidative burst, compromised antioxidant defenses causing electron leakage, and oxidative stress [23,24]. While low H_2_O_2_ levels act as signaling molecules in proliferation and immunity, excess H_2_O_2_ causes oxidative damage to proteins, lipids, and DNA. H_2_O_2_ is commonly used in scientific research as a tool to induce oxidative stress because it can penetrate biological membranes, subsequently causing cell dysfunction or death. *Eroicheir sinensis* is noted for its economic importance in Chinese aquaculture. In farming practice, the crab is particularly vulnerable to a series of environmental stressors and pathogens that exacerbate oxidative stress, undermining health and productivity. However, to date, there is still a lack of direct evidence regarding how the crab responds to oxidative stress. In this study, H_2_O_2_ was employed to elicit oxidative stress in the gill tissues of *E. sinensis*. We systematically analyzed: (1) antioxidant defense mechanisms, (2) regulatory dynamics of ion transport genes, and (3) cell apoptosis/autophagy, providing a comprehensive understanding of crustacean stress adaptation.

## 2. Materials and Methods

### 2.1. Animals and Treatments

Juvenile Chinese mitten crabs (one-year-old) were cultured in laboratory tanks (100 cm × 45 cm × 50 cm). The crabs were acclimated to lab conditions (pH 8.0 ± 0.2, DO > 6.0 mg/L, temperature 25 ± 1 °C) for two weeks. Commercial feed (Jiangsu Haorun Biotech. Co., Ltd., Taizhou, China) was administered twice daily, and one-third of the water volume was replaced every two days. Feeding was ceased one day before the experiment, and only healthy individuals that were in the intermolt stage and had intact appendages were selected for the experiments.

H_2_O_2_ solutions of varying concentrations (3, 6, 9, 12, and 15 mmol/L) were prepared by diluting 30% H_2_O_2_ in water. Uniformly sized healthy juvenile crabs (mean weight 13.34 ± 2.56 g) were subjected to the specified H_2_O_2_ concentrations for 96 h. A control group was placed with no H_2_O_2_ in water. Each group consisted of 90 individuals, distributed across three tanks. No food was provided to any of the groups during the experiment. The H_2_O_2_ solutions were refreshed daily. Following the treatment, nine juvenile crabs were randomly selected from each group at 24, 48, 72, and 96 h. These crabs were anesthetized via an ice-water bath prior to the collection of gills. Some samples were rapidly frozen in liquid nitrogen and stored at −80 °C for later analysis, while other samples were washed with PBS and soaked in 4% paraformaldehyde (Solarbio, Beijing, China) at room temperature for 24 h to perform paraffin embedding and sectioning.

### 2.2. Measurement of Antioxidant Parameters

The antioxidant capacity and oxidative stress status of gill tissues were systematically evaluated through a series of standardized assays. Gill tissues were precisely weighed and homogenized in ice-cold physiological saline (0.9% sodium chloride solution, mass-to-volume ratio 1:9) using a high-throughput tissue grinder. The homogenate was then centrifuged at 5000× *g* for 10 min at 4 °C to obtain the supernatant for analysis. Total protein (TP) concentration was measured using the Coomassie Brilliant Blue method (A045-2 kit), with absorbance readings at 562 nm, and the protein content was calculated. The total antioxidant capacity (T-AOC) was determined by the ABTS radical scavenging assay (A015-2-1 kit), measuring the reduction in absorbance at 593 nm after 5 min incubation at 37 °C. Superoxide dismutase (SOD) activity was evaluated using the hydroxylamine method (A001-1 kit) by monitoring the inhibition of nitrite formation at 450 nm. When the SOD inhibition rate reaches 50%, the corresponding enzyme is one SOD dynamic unit (U). Catalase (CAT) activity was measured via the ammonium molybdate method (A007-1-1 kit) by quantifying residual H_2_O_2_ at 405 nm after 60 s of reaction time at 37 °C. The activity of glutathione peroxidase (GPx) was assessed by monitoring the consumption of reduced glutathione at 412 nm (A005-1 kit). Malondialdehyde (MDA) concentration was determined using the thiobarbituric acid (TBA) method (A003-1-1 kit), by reacting MDA with TBA at 95 °C to generate a red product, with absorbance quantified at 532 nm. H_2_O_2_ concentration was analyzed using a ferrous oxidation–xylenol orange assay (S0038 kit), measuring the formation of a ferric complex at 520 nm after 30 min incubation at 25 °C. The results were normalized to total protein content. All experimental procedures were strictly performed in accordance with the manufacturers’ protocols for each respective assay kit. The commercial assay kits for TP, T-AOC, SOD, CAT, GPx, and MDA were purchased from Nanjing Jiancheng Bioengineering Institute (Nanjing, China). The H_2_O_2_ kit was acquired from Beyotime (Shanghai, China).

### 2.3. Histological Analysis

The gill tissue, initially fixed in 4% paraformaldehyde, was rinsed under running water for 4 h to remove residual fixative. Subsequently, a gradient ethanol process was employed for dehydration. Once dehydrated, they were embedded in paraffin wax and allowed to solidify. Sections of 5 μm thickness were prepared using a microtome Leica RM2235 (Leica, Nussloch, Germany). The slices were stained with hematoxylin and eosin (H&E) to highlight histological details. Finally, the sections were analyzed under an Leica DM4000 B LED light microscope (Leica, Hüllhorst, Germany) for histological details.

### 2.4. Quantitative Real-Time PCR (qPCR)

Total RNA was extracted from gills using a commercial kit (RNA-easy Isolation Reagent, R701, Vazyme Biotech, Nanjing, China) according to the manufacturer’s instructions. The concentration and purity of RNA were assessed by measuring the absorbance ratio at 260/280 nm using a NanoPhotometer-N50 (Implen, Munich, Germany). The cDNA was synthesized from 2 μg of total RNA using the PrimeScript™ RT reagent Kit (RR037Q, Takara, Beijing, China). Primers for related genes in this study were designed using the NCBI/Primer-BLAST tool (http://www.ncbi.nlm.nih.gov/tools/primer-blast/, accessed on 14 May 2024). Specific primer sequences and the corresponding GenBank accession numbers are detailed in Table 1. PCR reactions were performed on a CFX Opus 96 Real-Time PCR instrument (Bio-Rad, Hercules, CA, USA) with the following conditions: initial denaturation at 95 °C for 30 s, followed by 40 cycles of 95 °C for 5 s, 60 °C for 30 s, and a melting curve procedure. Each sample was prepared in triplicate. The relative mRNA expression levels of target genes to the internal reference gene were calculated using the 2^−ΔΔCT^ method.

### 2.5. Data Analysis

The results were presented as mean ± standard deviation (mean ± SD). Data were analyzed using SPSS Statistics 23.0 software (IBM, New York, NY, USA). A one-way ANOVA was employed for variance analysis, and Tukey’s test was used to examine significant differences between groups (*p* < 0.05). The normality of the distribution and the homogeneity of variances were assessed using the Shapiro–Wilk test and Levene’s test, respectively. GraphPad Prism 8.0 was used for statistical graphing.

## 3. Results

### 3.1. Gills Histological Observation

**Figure 1:**
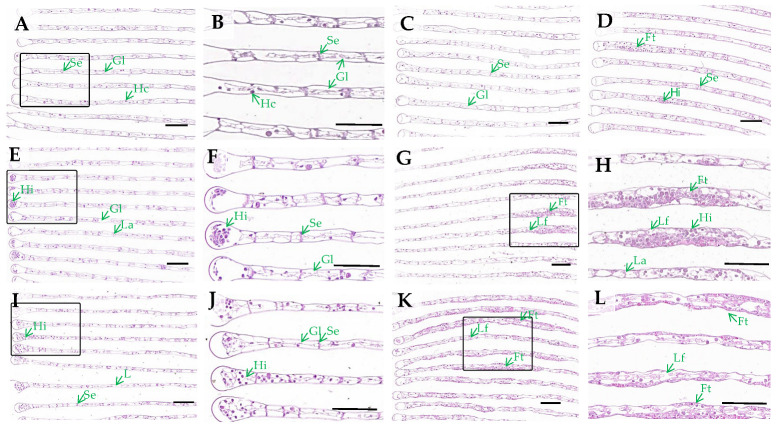
Effects of H_2_O_2_ exposure on gill morphology (HE-stained): (**A**,**B**) control; (**C**) 6 mmol/L + 24 h; (**D**) 6 mmol/L + 72 h; (**E**,**F**) 12 mmol/L + 24 h; (**G**,**H**) 12 mmol/L + 72 h; (**I**,**J**) 15 mmol/L + 24 h; (**K**,**L**) 15 mmol/L + 72 h. Gl, gill lumen; Se, septum; Hc, haemolymph cell; Hi, haemolymph cell increase; Ft: gill filament thickening. The boxed areas in (A,E,G,I,K) are magnified in (B,F,H,J,L), respectively. Scale bar = 50 µm.

Histological examination demonstrated concentration- and time-dependent alterations in the gill morphology of *E. sinensis* following H_2_O_2_ exposure. Control specimens exhibited well-arranged gill filaments with intact septum structure and a normal distribution of haemolymph cells (Figure 1A,B). At 6 mmol/L H_2_O_2_, no significant alterations were observed after 24 h (Figure 1C). However, after 72 h, slight thickening of gill filaments was detected (Figure 1D). More severe histopathological manifestations were evident at higher concentrations (12 and 15 mmol/L). At 24 h, a notable increase in haemolymph cells was observed, particularly at the tips of the gill filaments (Figure 1E,F,I,J). By 72 h, the gill filaments exhibited irregular thickening, septum degeneration, and extensive aggregation of haemolymph cells within the gill lumen (Figure 1G,H,K,L). Notably, both the severity of swelling and the affected area of gill filaments exhibited a positive correlation with increasing H_2_O_2_ concentrations at the 72 h time point, demonstrating clear dose-dependent pathological progression.

### 3.2. Impact of H_2_O_2_ Exposure on Antioxidant Capacity of Gills

**Figure 2:**
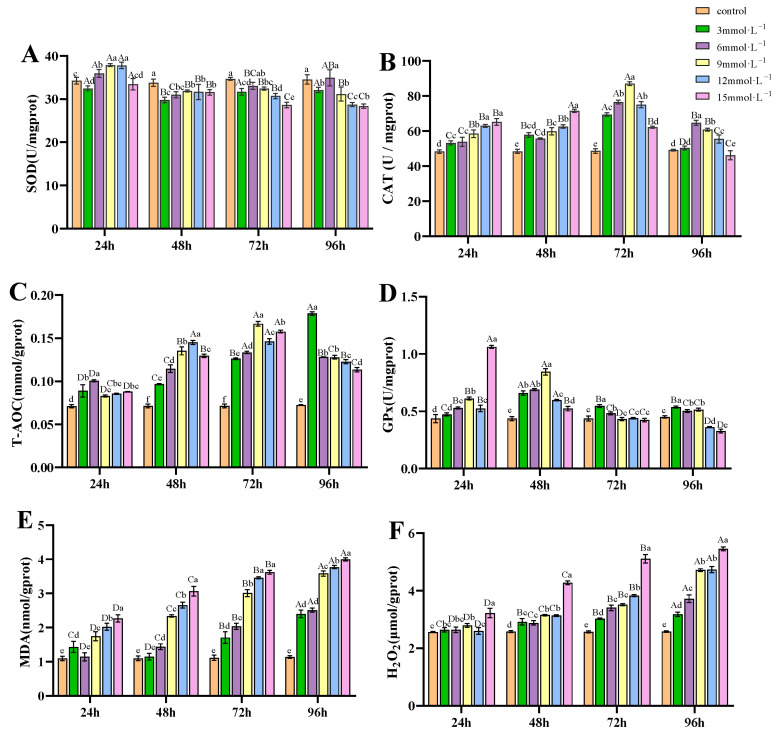
Effects of H_2_O_2_ exposure on antioxidant parameters in gills. (**A**) SOD; (**B**) CAT; (**C**) T-AOC; (**D**) GPx; (**E**) MDA; (**F**) H_2_O_2_. Distinct lowercase letters indicate significant differences at the same time point (*p* < 0.05), while distinct uppercase letters indicate significant differences across different time points within the same treatment group (*p* < 0.05).

Under H_2_O_2_ exposure, the SOD activities in the treatment groups of 6, 9, and 12 mmol/L at 24 h were higher than those in the control; while from 48 h, the SOD activities were significantly reduced compared to the control group (*p* < 0.05, Figure 2A). CAT activities showed an initial increase and then a decrease under the exposure of H_2_O_2_ (*p* < 0.05), with peak values observed at 72 h for the 3, 6, 9, and 12 mmol/L groups, and at 48 h for the 15 mmol/L group (Figure 2B). T-AOC in the treatment groups was significantly higher than in the control group (*p* < 0.05, Figure 2C), showing an increased trend from 24 to 96 h of exposure and peaking at 72 h, except for the 3 mmol/L group, which peaked at 96 h. GPx activities increased from 24 to 48 h in the 3, 6, 9, and 12 mmol/L groups, but decreased significantly below control at 96 h in the 12 and 15 mmol/L groups (*p* < 0.05, Figure 2D). After H_2_O_2_ exposure, the concentrations of MDA (Figure 2E) and H_2_O_2_ (Figure 2F) in gill tissues exhibited an overall increasing trend as time progressed.

### 3.3. Effects of H_2_O_2_ Exposure on the mRNA Expression of Ion Transport-Related Genes in Gills

**Figure 3:**
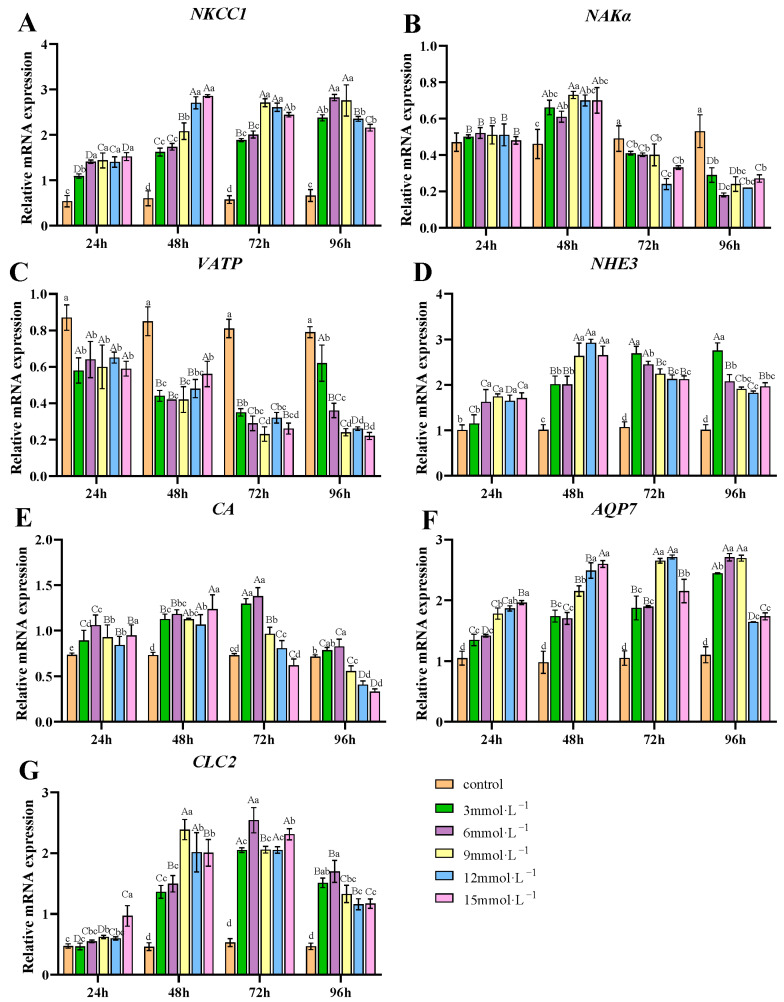
Effects of H_2_O_2_ exposure on the transcription of ion transport-related genes in gills. (**A**) *NKCC1*; (**B**) *NAKα*; (**C**) *VATP*; (**D**) *NHE3*; (**E**) *CA*; (**F**) *AQP7*; (**G**) *CLC2*. Distinct lowercase letters indicate significant differences at the same time point (*p* < 0.05), while distinct uppercase letters indicate significant differences across different time points within the same treatment group (*p* < 0.05).

H_2_O_2_ exposure influenced the transcription of ion transport-related genes in gill tissues (Figure 3). The transcription of *NKCC1*, *NHE3*, *AQP7*, and *CLC2* was upregulated after H_2_O_2_ exposure (Figure 3A,D,F,G). On the contrary, the transcription level of *VATP* was suppressed (*p* < 0.05) after H_2_O_2_ exposure (Figure 3C). The mRNA levels of *NKAα* initially increased across all treatment groups, peaking at 48 h, before subsequently decreasing. By 72 and 96 h, these levels were significantly lower than those observed in the control group (Figure 3B). The mRNA levels for *CA* initially rose, then declined, with the 3 and 6 mmol/L groups peaking at 72 h, whereas the 9, 12, and 15 mmol/L groups peaked at 48 h (Figure 3E).

### 3.4. Effect of H_2_O_2_ Exposure on the Transcription of Apoptosis-Related Genes in Gills

**Figure 4:**
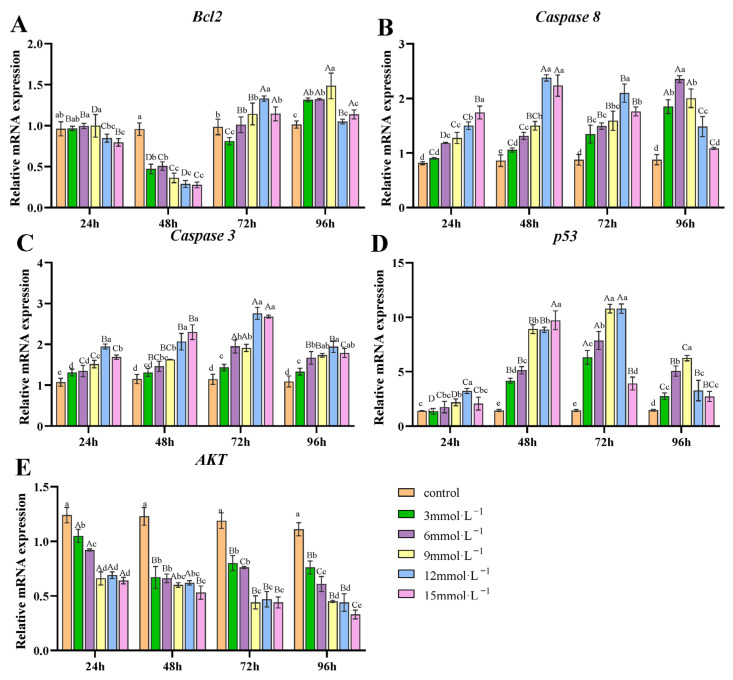
Effects of H_2_O_2_ exposure on the transcription of apoptosis-related genes in gills. (**A**) *Bcl2*; (**B**) *Caspase8*; (**C**) *Caspase3*; (**D**) *p53*; (**E**) *AKT*. Distinct lowercase letters indicate significant differences at the same time point (*p* < 0.05), while distinct uppercase letters indicate significant differences across different time points within the same treatment group (*p* < 0.05).

Under H_2_O_2_ exposure, *Bcl2* transcription was significantly inhibited at 48 h, showing values significantly lower than those of the control group (*p* < 0.05). However, at 72 and 96 h, there was a significant increase compared to 48 h (*p* < 0.05, Figure 4A). *Caspase-8* and *Caspase-3* transcription was significantly upregulated at all H_2_O_2_ concentrations and time points compared to the control (*p* < 0.05, Figure 4B,C). For *Caspase-8*, the 3, 6, and 9 mmol/L treatments showed a progressive increase in transcription over time, while in the 12 and 15 mmol/L groups, it reached its maximum at 48 h. For *Caspase-3*, its transcription peaked at 72 h. Similarly, the transcription of *p53* was significantly higher than the control (*p* < 0.05), reaching its maximum at 72 h (Figure 4D). The *AKT* transcription decreased significantly over time (*p* <0.05), with only a slight increase in the 3 mmol/L treatment group at 72 h (Figure 4E)

### 3.5. Effect of H_2_O_2_ Exposure on the Transcription of Autophagy-Related Genes in Gills

**Figure 5:**
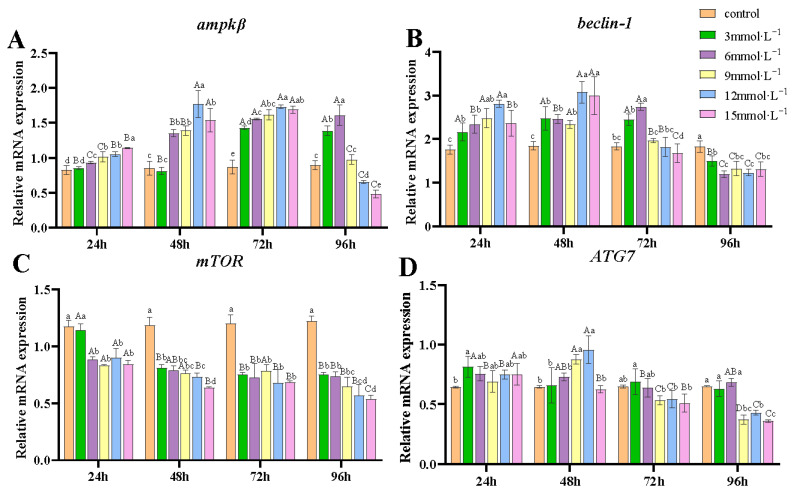
Effects of H_2_O_2_ exposure on the transcription of autophagy-related genes in gills. (**A**) *ampkβ*; (**B**) *beclin-1*; (**C**) *mTOR*; (**D**) *ATG7*. Distinct lowercase letters indicate significant differences at the same time point (*p* < 0.05), while distinct uppercase letters indicate significant differences across different time points within the same treatment group (*p* < 0.05).

At the H_2_O_2_ concentrations of 6, 9, 12, and 15 mmol/L, the transcription of *ampkβ* significantly increased from 24 to 72 h compared to the control (*p* < 0.05), while at the low concentration of 3 mmol/L, the upregulation occurred from 72 to 96 h (Figure 5A). The mRNA levels of *beclin-1* were significantly higher than those in the control group at 24 and 48 h (*p* < 0.05) (Figure 5B). By 96 h, *beclin-1* transcription was significantly suppressed compared to the control (*p* < 0.05). *mTOR* transcription was significantly downregulated under all conditions (*p* < 0.05) except at 3 mmol/L concentration after 24 h (Figure 5C). ATG7 transcription showed no significant difference from controls at 3 and 6 mmol/L (*p* > 0.05), but was significantly upregulated at 9 and 12 mmol/L after 48 h of exposure (Figure 5D). It is noteworthy that transcriptions of *ampkβ*, *beclin-1*, and *ATG7* were significantly downregulated at 96 h under exposure to 9, 12, and 15 mmol/L concentrations of H_2_O_2_.

### 3.6. Effect of H_2_O_2_ Exposure on the Transcription of Genes Related to MAPK Pathway in Gills

**Figure 6:**
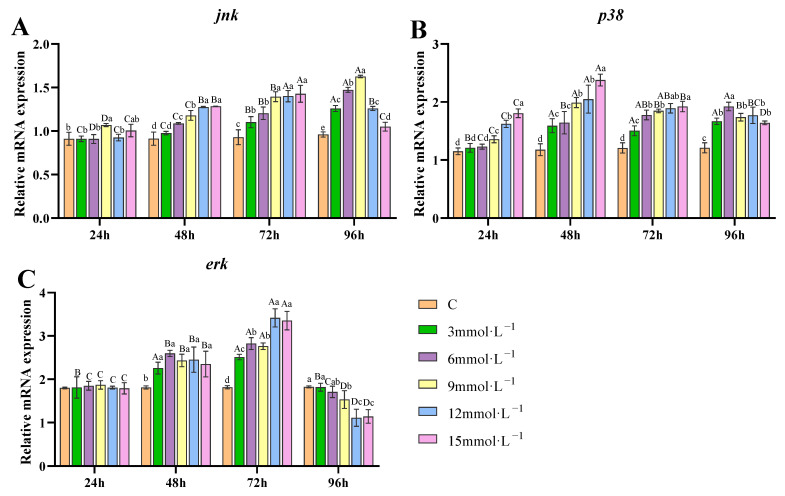
Effects of H_2_O_2_ exposure on the transcription of MAPK pathway-related genes in gills. (**A**) *jnk*; (**B**) *p38*; (**C**) *erk*. Distinct lowercase letters indicate significant differences at the same time point (*p* < 0.05), while distinct uppercase letters indicate significant differences across different time points within the same treatment group (*p* < 0.05).

After H_2_O_2_ exposure, mRNA levels of *jnk* exhibited a gradual increase from 48 to 96 h across all exposure concentrations. Although a slight decrease was observed in the 12 and 15 mmol/L treatment groups, levels remained markedly higher than the control group, with statistical significance (*p* < 0.05, Figure 6A). During the 96 h exposure, the transcription of *p38* exhibited a continuous increasing trend in the 3 and 6 mmol/L groups, with significant upregulation observed from 48 to 96 h. Meanwhile, *p38* transcription was markedly upregulated in the 9, 12, and 15 mmol/L groups after 24 h of H_2_O_2_ exposure, although it showed a declining trend at 96 h in the high concentration H_2_O_2_ groups (*p* < 0.05, Figure 6B). The transcription of *erk* was upregulated at 48 and 72 h under H_2_O_2_ exposure compared to the control. However, at 96 h, it decreased, showing levels lower than the control after 9, 12, and 15 mmol/L H_2_O_2_ exposures (*p* < 0.05, Figure 6C).

## 4. Discussion

### 4.1. Effects of H_2_O_2_ on Histological Changes and Antioxidant Capacity of Gills

In crustaceans, gills are a critical organ functioning primarily in respiration and ion regulation. These delicate structures can be particularly vulnerable to various environmental stressors, leading to physiological disorders or damage [29]. Consequently, gills are recognized as critical indicators of stress and disease. Romano and Zeng reported that ammonia-N exposure causes extensive infiltration of haemocytes, epithelial changes, disrupted pillar cells, and lamellae collapse in *Portunus pelagicus* gills [30]. Acute exposure to cadmium induced an increase in hemocytes in the gill lumen, with edema and even inflammatory foci in *Sinopotamon henanense* [13]. In this study, H_2_O_2_ exposure led to an increase in hemocytes, accompanied by enlarged gill lumen and degeneration of septa, showing hypertrophy of gill filaments at 72 h. Significant hemocyte infiltration within gill filaments serves as a characteristic histological marker of early inflammatory lesions [31]. This physiological state is considered an adaptive response to altered environmental conditions, functioning as a protective mechanism that prevents excessive toxin infiltration from water into gill tissues, thereby averting contamination of the hemolymph. However, this adaptation concurrently reduces the respiratory–excretory surface area of gills, ultimately compromising their functional capacity.

Oxidative stress occurs when the generation of ROS exceeds the scavenging capacity of the antioxidant defense system [32]. The ROS cascade typically begins with superoxide radical (O_2_^•−^) formation, which is then converted to hydrogen peroxide (H_2_O_2_) through SOD-catalyzed dismutation. H_2_O_2_ can subsequently generate hydroxyl radicals (^•^OH) via metal-catalyzed Fenton reactions, where H_2_O_2_ reacts with redox-active metals (Fe^2+^/Cu^+^) to generate ^•^OH [33]. The enzymatic antioxidant defense system plays a critical role in facing excessive free radicals to maintain cellular redox balance [34,35]. Common antioxidant enzymes such as SOD, CAT, and GPx can remove excess ROS and protect the body from oxidative harm [36]. The SOD isoenzymes, including cytoplasmic Cu/Zn-SOD and mitochondrial Mn-SOD, protect cells by neutralizing diffusible O_2_^−^ into H_2_O_2_ and O_2_, thereby blocking radical propagation. In this study, SOD activity exhibited a transient increase at 24 h of H_2_O_2_ exposure, demonstrating an initial adaptive response to stress. However, subsequent activity decline suggested possible H_2_O_2_-mediated enzyme inhibition, likely resulting from membrane-permeable H_2_O_2_ accumulation exceeding cellular detoxification capacity, as evidenced by elevated gill H_2_O_2_ levels. The underlying mechanism may involve excess H_2_O_2_ reducing catalytic Cu^2+^ in Cu/Zn-SOD to Cu^+^, which then participates in Fenton chemistry, generating damaging hydroxyl radicals and causing enzyme inactivation [37]. In contrast, Mn-SOD maintains relative stability due to the redox-inert nature of its manganese center, thereby avoiding Fenton reactions [38]. Unlike Mn-SOD, Cu/Zn-SOD appears more vulnerable under prolonged H_2_O_2_ stress. CAT mediates H_2_O_2_ decomposition to water and oxygen, while GPx reduces H_2_O_2_ to water using glutathione (GSH) as a substrate. In this study, CAT activity demonstrated a consistent rise from 24 to 72 h of exposure, while GPx activity exhibited a declining trend from 48 to 96 h. These inverse activity patterns reveal functional competition between GPx and CAT in H_2_O_2_ clearance beyond their complementary roles [39]. It is known that GPx has a much lower Michaelis–Menten constant value (*K_M_*) than CAT, which allows GPx to function efficiently at low concentrations of H_2_O_2_ (physiological levels), while CAT is significantly activated only when there is a high accumulation of H_2_O_2_ (under oxidative stress). The notable CAT upregulation confirms significant H_2_O_2_ accumulation in gill tissues, surpassing CAT’s high *K_M_* threshold. Concurrently, GPx’s dual-substrate requirement (H_2_O_2_ + GSH) makes it particularly susceptible to ROS overload, which oxidizes its catalytic selenocysteine residue and depletes GSH [40], inactivating enzyme function. T-AOC comprises the integrated function of enzymatic (SOD, CAT, GPx) and non-enzymatic (vitamins E/C, GSH, carotenoids, phenolics) antioxidants in neutralizing diverse free radicals, and is widely used to assess the overall antioxidant capacity in crustaceans under environmental stressors. Results demonstrated a rapid, significant increase in T-AOC levels from 24 to 96 h post-exposure compared to controls. The elevation likely represented a protective compensatory response to oxidative challenge, aimed at neutralizing the increased ROS to maintain cellular redox balance. Irregular changes in antioxidant enzyme activity were also observed in common carp and tilapia treated with H_2_O_2_ [24,41]. Moreover, similar results were observed in *E. sinensis* under oxidative stress induced by various environmental stressors. For example, SOD, CAT, and GPx activities in gills were increased after hypoxia stress, accompanied by increasing MDA content [42]. After cadmium (Cd) exposure, SOD, CAT, and GPx activities in the gills of *Charybdis japonica* showed an increased trend from 0.5 day to 3 days, but decreased after 6 days of exposure [43].

Both MDA and H_2_O_2_ have been utilized to assess the state of cellular oxidative stress. MDA, as a reactive aldehyde, is a byproduct of lipid peroxidation, generated when ROS (especially ^•^OH) attack polyunsaturated fatty acids (PUFAs) in cellular membranes [44]. H_2_O_2_ functions as both a redox signaling molecule at physiological levels and a toxic oxidant at pathological concentrations. While low levels regulate cell signaling via targeted oxidation, excess H_2_O_2_ overwhelms detoxification systems, causing oxidative damage [45]. Both MDA and H_2_O_2_ levels in this study revealed rising trends within the gill tissues of the treatment groups. Notably, the observed increase in H_2_O_2_ concentrations inversely correlated with SOD activity, suggesting reduced H_2_O_2_ generation via SOD, implying potential transmembrane diffusion of exogenous H_2_O_2_, and/or generation from alternative pathways such as peroxisomal fatty acid β-oxidation [46] or NOX-mediated production [47]. Excess H_2_O_2_ may generate additional ^•^OH through Fenton reactions, which continuously attack PUFAs to yield lipid hydroperoxides (LOOH) and ultimately MDA. Although GPx attempts to detoxify LOOH to non-toxic alcohols (LOH), its efficiency becomes progressively compromised due to ROS overload and GSH depletion. The intensified lipid peroxidation cascade ultimately leads to sustained elevation of MDA levels. Other exogenous stressors can also significantly elevate MDA and H_2_O_2_ levels. In *Scylla serrata*, lipid peroxidation and H_2_O_2_ were elevated in gills under salinity stress ranging from 10 ppt to 35 ppt [48]. Similarly, after a 14-day exposure to nitrite and sulfide, MDA and H_2_O_2_ contents increased significantly in *E. sinensis* gill tissues [49]. Collectively, evidenced by antioxidant/oxidant parameters and histopathological alterations, our study demonstrates that H_2_O_2_ can induce oxidative stress responses in gill tissues.

### 4.2. Effects of H_2_O_2_ Stress on the Ion Transport-Related Genes in Gills

Crustaceans maintain ion stability and fluid pH balance in complex environments through ion transport proteins and enzymes in gill tissues [50], which regulate intracellular and extracellular ion concentrations to support physiological functions. Key ion pumps like NKAα and VATP drive ion uptake and secretion by utilizing the energy released from ATP hydrolysis. VATP is responsible for transporting H^+^ either out of the cell or into organelles, while NKAα functions in maintaining the balance of Na^+^ and K^+^ both inside and outside cells [25]. Ion channels represent another class of membrane transport proteins that mediate selective ion movement, particularly Na^+^, Ca^2+^, and Cl^−^. Among these, NKCC1 co-transports ions across the cellular membrane in a ratio of 1Na^+^:1K^+^:2Cl^−^ [51]. The Na^+^/H^+^ exchanger (NHE) facilitates an exchange of Na^+^ and H^+^, maintaining the acid-base balance [52]. CA aids in the exchange of Na^+^ and Cl^−^ by catalyzing the hydration of CO_2_ in gill cells, providing counter ions H^+^ and HCO^3−^ [53]. CLC2 is responsible for regulating the transmembrane transport of Cl^−^. Our results show that the *VATP* transcription significantly decreased, while the *NAKα* transcription was only upregulated at 48 h and then downregulated at 72 and 96 h of exposure. The sustained downregulation of *VATP* suggests a suppression of proton transport into lysosomes, which may lead to a loss of lysosomal stability [54]. A study indicated that *VATP* transcript levels in *Macrobrachium amazonicum* gills were significantly downregulated under salinity stress relative to controls [51]. In contrast, the transient upregulation of *NKAα* at 48 h implies an immediate compensatory effort to maintain Na^+^/K^+^ homeostasis. The suppression of *NKAα* and *VATP* translation was possibly an attempt to conserve cellular energy under H_2_O_2_ stress by preventing ATP depletion. On the contrary, the mRNA levels of *NKCC1*, *NHE3*, *CA*, and *CLC2* showed a significant increase, indicating that H_2_O_2_ exposure stimulated the transcription of ion transport enzymes and may further promote ion movements in gills. Some reports showed similar changes in ion transport-related genes under stressors. Zhang et al. reported that the transcription of *NKA-α*, *NKCC1*, and *NHE* first increased and then decreased in the gills of *Portunus trituberculatus* under long-term ammonia stress [55]. Nan et al. reported that after 14 days of ammonia exposure in *Litopenaeus vannamei*, *NKA-α* and *NKA-β* genes were downregulated in the ammonia-exposed group compared to controls [56]. The transcription of *CA* increased and then decreased on *Macrobrachium nipponense* under nanoplastic exposure [57]. A study showed that the differentially expressed genes (DGEs) of antioxidant- and ion transport-related, e.g., NKA and GPx, were upregulated in the gills of *E. sinensis* under heavy metal cadmium toxicity [58]. In addition to maintaining ionic homeostasis, crustaceans also need to establish water balance within the body through aquaporins (AQPs) [59]. In *L. vannamei*, it was found that the transcription of *AQP7* significantly decreased with reduced salinity levels, hindering cellular water transport [60]. Our study revealed significantly elevated AQP7 mRNA levels following H_2_O_2_ exposure, suggesting enhanced water transport activity in gill tissues. This may contribute to the observed gill filament swelling. It is reported that oxidative stress modulates ion transport through both direct and indirect mechanisms. Direct effects involve oxidative modification of critical amino acids, such as cysteine thiol groups, in ion channels, inducing conformational changes that alter their function [61]. Indirect effects occur via (1) ROS-dependent phosphorylation that post-translationally regulates channel activity [62], (2) membrane lipid peroxidation disrupting the structural integrity of channel microenvironments, and (3) activation of NF-κB and Nrf2 signaling cascades that transcriptionally modulate ion transport-related gene expression profiles [63]. These findings demonstrate that oxidative stress dynamically regulates both ion and water transport systems in crustacean gills, possibly leading to impaired osmoregulatory homeostasis.

### 4.3. Effects of H_2_O_2_ Exposure on Apoptosis and Autophagy of Gill Tissues

Oxidative stress can trigger cellular apoptosis, a programmed cell death process critical for maintaining cellular homeostasis [64]. Apoptosis is promoted through two pathways: the extrinsic pathway, which is initiated by direct activation of caspase-8 [65]; and the intrinsic pathway, triggered by mitochondrial dysfunction, leading to cytochrome c release and subsequent caspase activation [66]. Both pathways ultimately converge on the cleavage of executioner caspase-3/7 to induce cell death [67]. In this study, mRNA levels of *caspase-8* were significantly upregulated in a dose- and time-dependent manner at 3, 6, and 9 mmol/L concentrations, suggesting the activation of extrinsic apoptosis. *Caspase-3* transcripts were significantly upregulated in all H_2_O_2_ treatment groups and reached their peak at 72 h, further confirming the activation of apoptosis. Similar results were reported in previous studies, where the mRNA expression patterns of *caspase-8*/*3* were upregulated in the gills of both *Sinopotamon henanense* after H_2_O_2_ exposure [68] and *L. vannamei* under NH_4_Cl exposure [69]. Bcl2 is a well-characterized anti-apoptotic protein that suppresses programmed cell death by regulating the mitochondrial pathway of apoptosis [70]. Following 48 h of H_2_O_2_ exposure, significant suppression of *Bcl2* transcription suggests the initiation of the mitochondrial pathway of apoptosis. By 72 h, an increase in *Bcl2* transcription, particularly in treatment groups at concentrations of 12 and 15 mmol/L, may reflect the cells’ adaptation to sustained oxidative stress or an attempt to enhance their survival prospects by elevating *Bcl2* transcription. The specific mechanisms require further investigation in future studies. Guan et al. found that abamectin could promote apoptosis in *P. clarkii* based on the downregulation of *Bcl2* and upregulation of *caspase-3* [71]. Additionally, Liu et al. demonstrated that the downregulation of *Bcl2* and upregulation of *caspase-3* induced by LPS stress can be reversed by adding dietary glutathione [72]. Furthermore, oxidative stress may activate specific transcription factors, such as *p53*, which induces apoptosis by triggering key apoptotic genes, including the mitochondrial proapoptotic factor Bax and the death receptor Fas [73,74]. When exposed to persistent organic pollutants (HBCD or BDE-47), apoptosis could be triggered by oxidative stress through the transcriptional upregulation of *p53* in the gills of *Macrophthalmus japonicas* [20]. Sun et al. reported that hypoxia-induced oxidative stress can cause apoptosis in *M. nipponensem*, which was proved by the increase in *caspase-3* mRNA expression and the upregulation of *p53* at transcript and protein levels [75]. In this study, the upregulation of *p53* transcripts in gill tissues may induce the transcription of proapoptotic genes to accelerate apoptosis. Furthermore, Akt, a serine-threonine kinase, is crucial for cellular proliferation and survival, as it inhibits apoptosis by phosphorylating various target molecules [76]. Following 48 h of exposure to nitrite or ammonia, transcription of *Akt* was notably downregulated in the gills of *L. vannamei* [77]. A similar result was identified by Huang et al., who found that, under prometryn stress, the transcription of *Akt* was decreased while *caspase-3* increased at 48 h [78]. In this study, *Akt* decreased significantly over time after H_2_O_2_ treatment, indicating that apoptosis in the gills of *E. sinensis* may be associated with the inhibition of the PI3K–Akt signaling pathway [79]. Based on the transcription of these genes, we revealed that H_2_O_2_ may promote cell death in the gills of *E. sinensis* through the apoptosis program. 

Autophagy is another cellular strategy for combating stressful environments, particularly under conditions of nutrient or energy deficiency. Increasing evidence suggests that autophagy plays a pivotal role as a sensor in redox signaling during cellular responses [80]. Mild oxidative stress activates the autophagy pathway as a self-protective mechanism. However, severe oxidative stress compromises this process, thereby accelerating cell apoptosis and necrosis [81]. The autophagic process is governed by an intricate network of molecular regulators, including Atg family proteins and lysosomal degradation enzymes. Beclin-1 is a crucial mediator of autophagy, which facilitates the initial formation of phagophore membranes that develop into autophagosomes. The maturation of these autophagic vesicles relies on specialized protein conjugation mechanisms, particularly those involving Atg7 and the LC3 protein. Moreover, cellular energy sensors, specifically the AMPK and mTOR pathways, serve as central regulators that modulate autophagic activity under stress conditions. It has been reported that elevated levels of ROS are associated with increased expression of autophagy regulatory factors, such as *beclin-1* and *ATG7* [82]. Upregulation of beclin-1 enhances the formation of autophagosomes [83]. However, Bcl2 can bind to beclin-1, inhibiting its activity to prevent excessive autophagy and thus protect cells from damage [84], modulating the balance between the two critical pathways of autophagy and apoptosis. In *M. nipponense*, hypoxia stress significantly increased the mRNA and protein levels of beclin-1 [85]. Additionally, ROS can activate *ampk*, which in turn promotes autophagy by inhibiting the transcription of *mTOR* [28]. Hypoxia induced antioxidant responses, which subsequently trigger autophagy in hepatocytes by upregulating *beclin-1*, *ATG7*, and *ATG8* at both transcriptional and protein levels in *M. nipponense* [86]. *Spiroplasma eriocheiris* infection can induce autophagy accompanied by the increased transcription of *ATG7* in *E. sinensis* [87]. In our study, the *beclin-1* transcription was upregulated from 24 to 72 h of exposure but downregulated at 96 h. The transcription of *ATG7* increased slightly after 48 h of exposure to H_2_O_2_ and was then downregulated. Similarly, *ampkβ* showed an increased trend after H_2_O_2_ exposure from 24 to 72 h, but its levels decreased at 96 h under high concentrations of H_2_O_2_ (9, 12, and 15 mmol/L). The upregulation of these genes indicated that mild H_2_O_2_ exposure induced autophagy in gill cells, an adaptive response mechanism. However, the downregulation suggested that high concentrations and continuous exposure to H_2_O_2_ may inhibit autophagic functions.

The MAPK signaling pathways, including ERK, JNK, and p38, play a pivotal role in cellular apoptosis and autophagy [88]. Under stress conditions, MAPKs integrate various signals through transcription-dependent and independent mechanisms based on the cellular environment and type, ultimately maintaining the balance between cell survival and death [89]. Increased ROS leads to the activation of stress-responsive kinases such as JNK and p38 MAPK, which phosphorylate and stabilize p53. p53 induces apoptosis by transcriptionally activating proapoptotic genes such as Bax and PUMA to disrupt mitochondrial integrity and FAS to initiate extrinsic apoptosis. Additionally, it promotes oxidative stress through PIGs and FDXR, amplifying oxidative stress and apoptotic signaling [90]. Guyton et al. discovered that H_2_O_2_ is a key factor in the activation of MAPKs by triggering various receptors [91]. Peng et al. discovered that cadmium could induce antioxidant response in *L. vannamei*, leading to an increased transcription of *JNK*, *p38*, and *ERK* [92]. Our study observed that exposure to H_2_O_2_ upregulated *JNK*, *p38*, and *ERK* in the gill tissues, suggesting a critical role for the MAPK signaling pathway in the response to oxidative stress.

It has been proven that autophagy and apoptosis can be simultaneously regulated and occur within the same tissue. For instance, hypoxia caused severe oxidant stress, activated apoptosis [75] and autophagy [86] in the hepatopancreas of *M. nipponense*. Similar results were also found in *E. sinensis* under copper ion stress [93] and sustained hepatopancreatic necrosis disease [94]. Both scenarios are characterized by severe oxidative stress. Research indicates that apoptosis and autophagy exhibit mutual inhibition, though their exact regulatory interplay remains unclear. Both processes share key factors like Beclin-1 and Bcl2, which act as molecular switches [95]. Bcl2 suppresses apoptosis by inhibiting Bax/Bak while also binding and inactivating Beclin-1 to block autophagy. This study further demonstrated an inverse correlation between the expression trends of *Bcl2* and Beclin-1. The dual regulatory roles of the AMPK and MAPK pathways in this process are particularly noteworthy. AMPK promotes cell survival by activating autophagy to remove damaged organelles and mitigate oxidative damage, while MAPK subfamilies (such as JNK and p38) drive proapoptotic signaling to eliminate severely compromised cells. This mechanism likely plays a crucial role in the reparative capacity of gill tissues under stress.

## 5. Conclusions

Our study investigated the multidimensional adaptive response mechanisms of *E. sinensis* to oxidative stress induced by various concentrations of H_2_O_2_ in gill tissues. Initially, the crabs enhanced their antioxidant defense by upregulating key antioxidant enzymes (SOD, CAT, GPx) and increasing T-AOC level, which helped mitigate initial oxidative damage. The stress also challenged the gills’ ion regulation capabilities, requiring adaptive gene transcription changes to maintain cellular homeostasis. However, excessive exposure led to an overwhelmed antioxidant system, peroxide accumulation, gill inflammation, and ion-regulatory dysfunctions. Furthermore, H_2_O_2_ triggered the AMPK and MAPK signaling pathways, leading to autophagy and apoptosis. In summary, this study provides insights into the oxidative stress responses of crabs to H_2_O_2_ and highlights the critical functions of the gill tissues under such conditions.

## Figures and Tables

**Table 1:** Sequences of primers used in qPCR.

Gene	Description	Primer Sequence (5′-3′)	Product Length (bp)	GenBank Accession Number
*NHE3*	Na^+^/H^+^ exchanger 3	CGCAACCCACCGTAGCTCAT TGCTCCGTCTACCGCATCCT	113	SRR769751
*NKAα*	Na^+^/K^+^-ATPase subunit α	TCTGCTTCATCGCCTACTCCA AGGACTCCATGATACGCGAAC	153	KC691291.1
*CA*	carbonic anhydrase	CTCGCAGTTCCACTTCCACT CGTGTTTCAATGCCTCGTCC	122	XM_050884291.1
*NKCC1*	Na^+^-K^+^-2Cl^−^ cotransporter-1	TGCCTCAGGGTCTTGACTACTCC GCCTCACTGTCTGTTCCGTCT	154	MF062032.1
*VATP*	V-type proton ATPase subunit d1-like	AGTTGATGCCTAAATGCC TCGTCCAAGTCCTGCTC	154	XM_050847128.1 [25]
*AQP7*	aquaporin 7	CACTCTCGTTGGTGGATGGG GTGGAGGTGTCCTGGTGC	201	XM_050868012
*CLCN2*	Chloride channel protein 2	CAGCCCTCAAGCAAACA GGAGGCGATGGCTATTT	192	XM_050870418.1 [25]
*jnk*	c-Jun N-terminal Kinase	TACAGTAGAGGTGGGCGACA TAGGCTCGCTTGGCATGAG	180	KC900087
*p38*	p38 mitogen-activated protein kinase	AAGATCACCAGCGATGAGGC TGCTAGGTAGGGATGGGCAA	183	KF582665.1
*erk*	extracellular signal-regulated kinase 2	CGCGAGTTGCAGATCCAGAA CAAGGGGCGATTGGACAACA	170	GU002542.1 [26]
*Caspase-8*	Cysteine-aspartic acid protease 8	TGGAGCGTCATGGTTCAGAC CAGACAAGCCACCACTGCTA	161	AKS36884.1
*Caspase-3*	Cysteine-aspartic acid protease 3	GCTGCTAAGCCAGTAGGCTG CATATTGCCCACGCTCTGGAA	130	MH183147.1
*Bcl2*	bcl-2-like protein1	AAAAGGAACCTGTGGCGTCT GAGACGGCGAGCCTTGATAA	209	XM_050860189.1
*P53*	cellular tumor antigen p53-like	TCGACATGGAAGGGAAGCAC CTGACTTCAAACGGCACAGC	139	JQ613218.1
*AKT*	AKT-threonine/serine protein kinase	CAAGATCCTGCGCAAAGACG CATGACGAAGCAGAGACGGT	148	KY412800.1 [27]
*ATG7*	ubiquitin-like modifier-activating enzyme ATG7	GCTCTGGGCTTTGACTCCTT TCGTGTGTGGAATTCCCTGG	167	MT543027.1
*ampkβ*	5′-AMP-activated protein kinase subunit beta-1	CAATCGTTGACCTCCCAGAA ACTTCCCTTTCCTTCCCAGAG	232	MK676045.1
*mTOR*	serine/threonine-protein kinase mTOR-like	AGAAGCTGCATGACTGGGAC CGGTCACACGACACACTGTA	148	XM_050855996.1 [28]
*Beclin-1*	beclin-1-like protein	GCCCATATACTGTGGCGAGG CCAGGTCAAAGAGCCCAGTT	176	MH173046.1
*UBE*	internal standard gene	TTGCGTTCACAACTCGTATCTACC GTCCGTGAGGAGGGAACAGA	137	HQ436509

## Data Availability

The original contributions of this study are detailed within the manuscript. Further inquiries should be directed to the corresponding author.
